# Influence of Dopaminergically Mediated Reward on Somatosensory Decision-Making

**DOI:** 10.1371/journal.pbio.1000164

**Published:** 2009-07-28

**Authors:** Burkhard Pleger, Christian C. Ruff, Felix Blankenburg, Stefan Klöppel, Jon Driver, Raymond J. Dolan

**Affiliations:** 1 Wellcome Trust Centre for Neuroimaging at UCL, London, United Kingdom; 2 Department of Cognitive Neurology, Max Planck Institute for Human Cognitive and Brain Sciences, Leipzig, Germany; 3 UCL Institute of Cognitive Neuroscience, London, United Kingdom; 4 Laboratory for Social and Neural Systems Research (SNS), Institute for Empirical Research in Economics, University of Zurich, Switzerland; 5 Department of Neurology and Bernstein Center for Computational Neuroscience, Charité, Berlin, Germany; 6 Department of Psychiatry and Psychotherapy, University of Freiburg, Freiburg, Germany; Mount Sinai School of Medicine, United States of America

## Abstract

Reward-related dopaminergic influences on learning and overt behaviour are well established, but any influence on sensory decision-making is largely unknown. We used functional magnetic resonance imaging (fMRI) while participants judged electric somatosensory stimuli on one hand or other, before being rewarded for correct performance at trial end via a visual signal, at one of four anticipated financial levels. Prior to the procedure, participants received either placebo (saline), a dopamine agonist (levodopa), or an antagonist (haloperidol). Principal findings: higher anticipated reward improved tactile decisions. Visually signalled reward reactivated primary somatosensory cortex for the judged hand, more strongly for higher reward. After receiving a higher reward on one trial, somatosensory activations and decisions were enhanced on the next trial. These behavioural and neural effects were all enhanced by levodopa and attenuated by haloperidol, indicating dopaminergic dependency. Dopaminergic reward-related influences extend even to early somatosensory cortex and sensory decision-making.

## Abstract 2

This pharmacological fMRI study shows that during reward-based sensory decision-making, dopamine is crucially involved in reward-related modulation of human primary sensory cortex.

## Author Summary

The rewards one receives during decision-making has a profound impact on learning. Much recent interest has focused on the role of the neurotransmitter dopamine in the basal ganglia for influencing learning and behaviour. Here, we ask whether reward can influence low-level sensory processing, for instance in primary sensory cortex, and how dopamine mediates this process. We show in humans that dopamine level, as manipulated with a dopamine agonist and antagonist in a double-blind placebo-controlled design, is involved in reward modulation of primary somatosensory cortex. Higher anticipated reward improved tactile decisions, and receipt of visual reward signals reactivated primary somatosensory cortex for the judged hand as measured using functional neuroimaging. After receiving a higher reward on one trial, somatosensory activations and decisions were enhanced on the next trial, suggesting that reward outcome provides a form of teaching signal that may be fed back to task-relevant sensory cortex. All these behavioural and neural effects of reward on somatosensory decision-making were strongly modulated by the availability of dopamine as the mediating neurotransmitter. These findings raise the tantalising new possibility that reward manipulations in conjunction with dopaminergic drugs might be used to enhance pathologically deficient or lapsed sensory processes, analogous to how rewards can be used to shape or correct behaviour.

## Introduction

A role for dopamine in Pavlovian and instrumental learning, as well as in consolidating plastic changes in corticostriatal pathways, is well established [1],[2]. Although research on reward has focused on learning, there is growing interest in a possible reward-mediated modulation of perception and sensory decision-making [3]–[5]. However, it remains unclear whether effects of reward on human sensory processing are influenced by dopamine.

**Figure 1:**
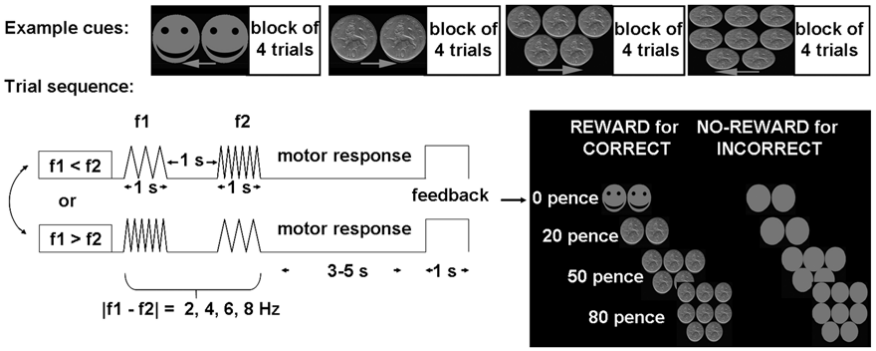
Schematic of somatosensory frequency discrimination task and reward paradigm. There were four possible reward magnitudes (0, 20, 50, and 80 pennies per correct trial), grouped into miniblocks of four trials. A distinct visual cue signalled the onset of a miniblock (four different examples shown in the top row). This cue indicated via a visual icon the potential reward for each of the next four trials, and also whether the right or left index finger was rewarded (conveyed by arrow below the icons). At lower left, a schematic sequence of events is shown for one trial. Both index fingers were simultaneously stimulated electrically, twice in succession; participants discriminated the frequency of the two successive stimuli (f1 and f2, f = frequency) for the hand arrowed by the preceding miniblock cue (see top row examples). After the second stimulus (f2), participants had to indicate whether f1 or f2 was higher (or lower, counterbalanced across participants), by pushing a pedal with both feet once for f1 or twice for f2. Three to 5 s after offset of the second electrical stimulus (randomly jittered in steps of 1 s), and thus 6 to 8 s after onset of the first, they received visual reward or no-reward feedback via icons (see eight different examples in box at bottom right). This jittered separation of reward delivery, via visual feedback at trial end, from the preceding somatosensory stimulation/discrimination allowed us (together with performance-contingent rewarded or nonreward outcomes, and the different reward magnitudes) to isolate hemodynamic responses specific to delivery of different rewards; see Materials and Methods.

Here, we examined possible dopaminergic modulatory influences on neural activity in human primary somatosensory cortex (PSC) and on sensory decisions. We exploited a new somatosensory paradigm for which we recently showed that increased financial rewards not only improve sensory performance, but also modulate PSC at the point of reward delivery, even when the financial reward is presented only visually [6]. To examine any contribution of dopamine to reward modulation of somatosensation, we now combine the sensory decision-making paradigm with concurrent functional magnetic resonance imaging (fMRI) (see Materials and Methods, and Figure 1) in the context of both agonist and antagonist dopaminergic pharmacological manipulations.

In a placebo-controlled, double-blind, fully randomized design, participants received pills comprising either 100-mg levodopa, 2-mg haloperidol, or placebo (see Materials and Methods, and Figure S2). Levodopa is well established for increasing brain dopamine levels, as commonly used as therapy for Parkinson disease [7]. Haloperidol is an antidopaminergic drug (selective D2 receptor antagonist), frequently used to treat psychosis [8].

## Results

### Influences of Pharmacology and Reward on Somatosensory Decision-Making

**Figure 2:**
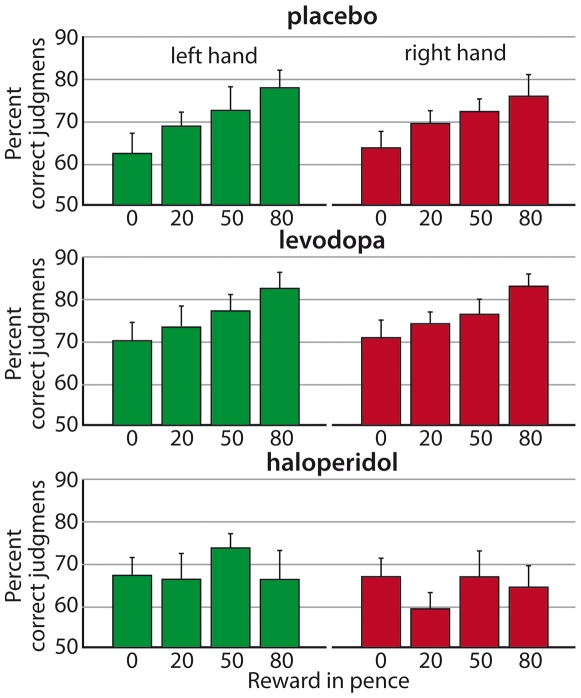
Percent correct judgments for different reward magnitudes under placebo, levodopa, or haloperidol. Results are shown separately for the right (red) and left (green) index fingers, plotting group means±s.e.m. For placebo (top row), we found a monotonic effect of increasing potential reward leading to increasing proportion of correct sensory decisions. This effect was more pronounced for levodopa (middle row), but attenuated, and indeed eliminated, for haloperidol (bottom row). These findings indicate that the impact of the anticipated amount of financial reward on tactile decision accuracy is modulated by dopaminergic influences.

We found a clear impact of dopaminergic modulation on somatosensory decisions. During scanning, across all reward levels, percentage correct somatosensory judgments comprised 70.4% of trials for the placebo group, increased to 76.3% for the levodopa group, and reduced to 66.4% for the haloperidol group. In terms of the specifics of reward effects, within the present placebo group (and in accord with our recent nonpharmacological study [6]), increased potential reward led to enhanced accuracy of sensory decisions (Figure 2, top row; linear parametric effect of reward level *F*_(1,9)_ = 9.99, *p* = 0.012) for judgments about the left or right hand (no significant main effects or interactions with factor of side, all *p*>0.99). This effect of reward on somatosensory decisions was affected by our pharmacological manipulation (see Figure 2, comparing different rows), leading to a significant interaction between drug group and reward level (*F*_(2,27)_ = 3.81, *p* = 0.035). Again, this outcome did not depend on the hand judged (no significant main effects or interactions involving side, all *p*>0.5).

Planned comparisons for the impact of the different drugs showed that under levodopa (middle row in Figure 2), overall accuracy was significantly higher than for placebo (*F*_(1,18)_ = 5.68, *p* = 0.028), whereas higher reward levels still systematically increased discrimination accuracy (*F*_(1,9)_ = 19.16, *p* = 0.002). By contrast, haloperidol (bottom row in Figure 2) not only attenuated the effect of reward level relative to placebo (*F*_(1,18)_ = 5.32, *p* = 0.03), but actually eliminated the impact of reward level (*F*_(1,9)_ = 0.03, *p* = 0.85, n.s.). Thus, these data show that for sensory decisions involving the left or right hand, agonist and antagonist dopamine manipulations enhance accuracy or reduce reward-related effects on somatosensory discrimination performance, respectively.

We next examined the fMRI data acquired concurrently with task performance for all three pharmacological groups, analyzing these with standard approaches (SPM5 software, see Materials and Methods for details). To anticipate, we observed effects of the dopaminergic manipulations on brain activity related to reward and to somatosensory processing that corresponded with the effects on somatosensory decisions reported behaviourally above, and that shed light on the neural mechanisms involved.

### The Brain Network Involved in the Somatosensory Decision Task

During the somatosensory discrimination phase of each trial, we found activation of a task-related network of brain areas including PSC and secondary somatosensory cortices/parietal ventral cortex, as well as prefrontal cortex (PFC), supplementary motor area (SMA), premotor cortex (PMC), posterior parietal cortex (PPC), insula, caudate nucleus, and striatum in both hemispheres (see Text S1 and Table S1). This accords with the involvement of similar areas for related somatosensory tasks in other work [6],[9].

### Influence of Reward and Drugs on Reward Regions and on PSC during Reward Delivery

**Figure 3:**
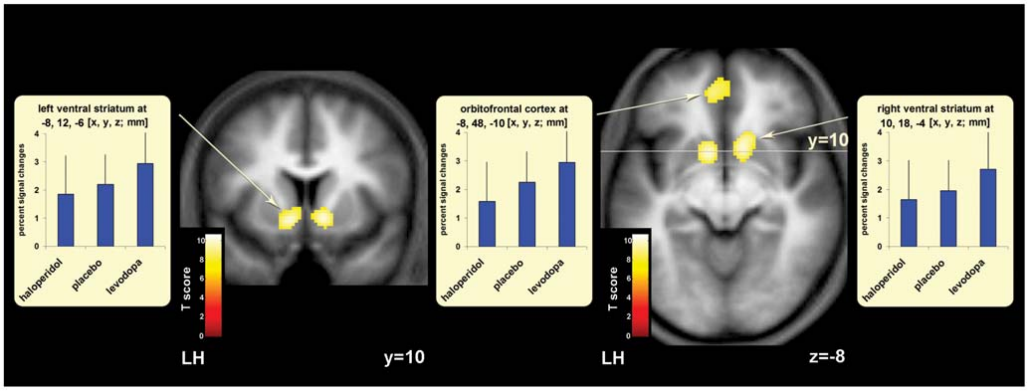
Cortical regions where BOLD responses to reward were affected by central dopamine level. BOLD responses to reward (all visual feedback events that indicated reward delivery, compared with the visual nonreward feedback events, at trial end) were affected by central dopamine level (linear parametric effect of the three drugs “haloperidol<placebo<levodopa,” for drug-by-reward interaction thresholded at *p* = 0.05 FWE-corrected; left hemisphere [LH]). This test revealed higher activity in ventral striatum and orbitofrontal cortex for rewarded trials when participants were pretreated with levodopa (i.e., a drug that increases central dopamine levels) and lower activity there when participants were given haloperidol (i.e., a dopamine receptor antagonist), for reward versus nonreward trials. Both the ventral striatum and orbitofrontal cortex, well established as key areas of the brain's reward system, are thus susceptible to changes in central dopamine level. See Table S2a for coordinates, *p*-values. and T-scores.

To identify brain regions where dopamine level specifically influenced reward-related activation, we next focused on blood oxygen level–dependent (BOLD) signals during the visual reward outcome presented at trial end (see Figure 1), in the absence of somatosensory stimulation. When testing for the interaction of group and outcome (rewarded versus nonrewarded) at trial end, we observed greater differential BOLD signal in ventral striatum and orbitofrontal cortex (OFC). Both these regions showed a reliable group-by-reward interaction, attributable to an enhancement of the reward-related signal for the levodopa (increased central dopamine) group and an attenuation of reward effects for the haloperidol (D2 receptor antagonist) group, relative to intermediate reward-related responses seen under placebo (see Figure 3, plus Table S2a). Thus, BOLD signals in two key regions implicated in reward, (i.e., ventral striatum [10] and OFC [11]), showed a response profile at reward delivery that clearly depended on dopamine level.

**Figure 4:**
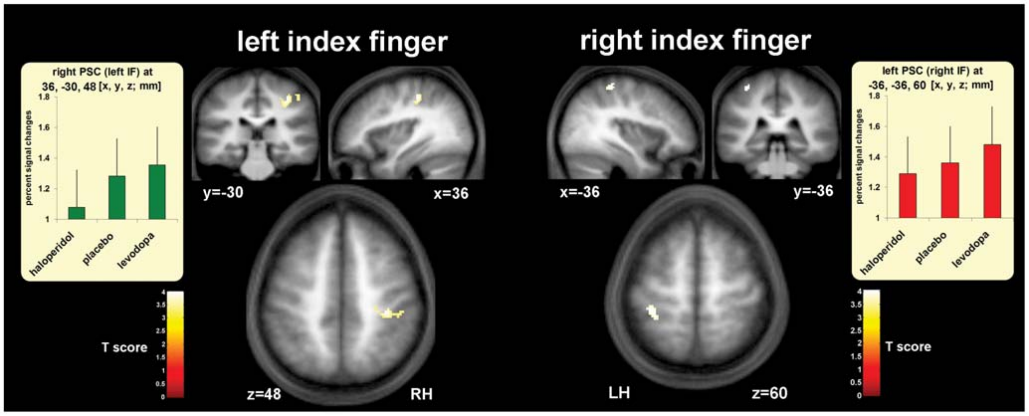
Cortical regions within PSC where BOLD responses to reward were affected by central dopamine level. In those trials in which either the left or right index finger (IF) had been rewarded for correct judgment, regions within contralateral PSC (including BA1, BA2, and BA3b) showed changes in BOLD responses reflecting an interaction between central dopamine levels and reward, at the time point corresponding to when financial reward or nonreward was delivered visually (drug-by-reward interaction thresholded at *p* = 0.05, FWE-corrected, see Materials and Methods for further details; LH, left hemisphere; RH, right hemisphere). Reactivation of contralateral PSC by visual reward-delivery (Table S2b; peaking at −34, −40, 60 for left PSC; and 36, −30, 50 for right PSC) increased with increased central dopamine levels; an effect comparable to the drug effect seen in ventral striatum and OFC (cf. Figure 3). In contrast to the positive results at the time point corresponding to visual reward delivery, we found no parametric effect of reward level on PSC for the earlier discrimination phase of trials, when contrasting correct minus incorrect trials there (see Figure S1, and Text S1). This indicates that the effects on PSC shown here (and in Figure 5) reflect actual reward delivery via the visual feedback, rather than other factors such as sensory attention during the stimulation; see also [6].

The same comparison (interaction of drug group with reward versus no reward) also revealed dopamine-related influences on activity within PSC itself (see Table S2a). Note that this reward-dependent somatosensory activation was expressed at a time point corresponding to the delivery of visual rewards at trial end. We confirmed that these activations originated from PSC, by restricting our examination of BOLD signals to primary somatosensory areas BA1, BA2, and BA3b [12], as defined by a computerized atlas based on cytoarchitectonic data [13] (see Materials and Methods for further details). These analyses confirmed a reward effect (relative to nonrewarded trials) in PSC, at the time point corresponding to visual reward delivery. Moreover, this somatosensory effect also depended on dopamine level, as manipulated here pharmacologically (see Figure 4, plus Table S2a and S2b).

The involvement of PSC in this impact of reward, at the reward delivery point during a sensory decision task, accords with our recent nonpharmacological findings [6]. That previous study also showed that visually signalled financial rewards can “reactivate” PSC in the context of a somatosensory-discrimination task. This suggests that reward outcome provides a form of teaching signal that may be fed back to task-relevant sensory cortex. The present data now show that the effectiveness of reward in influencing PSC in this way depends on dopamine, as evident in our new demonstration that the impact on somatosensory cortex itself is enhanced under levodopa and attenuated under haloperidol (see Figure 4, Table S2a and S2b), analogously to the effects we found also for more classic reward-related regions (see Figure 3).

Importantly, these dopaminergic influences on reward effects in somatosensory cortex were expressed specifically in the PSC that was required for the preceding decision that led to the reward. Separate analyses of trials in which the left or right index finger had been judged revealed that only somatosensory cortex contralateral to the currently judged hand was affected by reward delivery and by drug group in this way (Figure 4 and Table S2b; peak at *xyz* = −36, −36, 60 for left PSC when judging the right hand; and at 36, −30, 48 for right PSC when judging the left hand). This underlines that the dopaminergic reward influences were indeed specifically expressed only in the portions of PSC that were relevant for correct performance of the preceding task.

**Figure 5:**
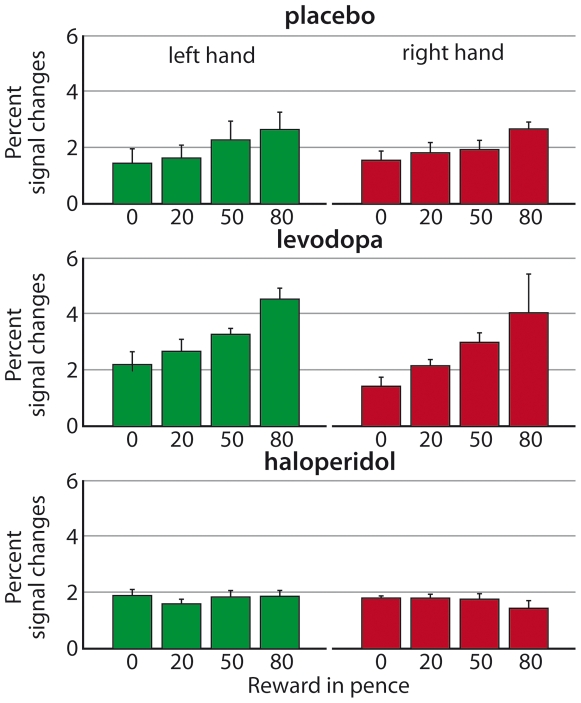
Percent signal changes in PSC corresponding to the time point of visual reward delivery. The ROIs contralateral to the rewarded right (red) or left (green) index finger are shown in separate rows for the three drugs groups (group mean±s.e.m.). For placebo (top row), reactivation in PSC by visual reward delivery increased systematically with financial magnitude of the reward received. Under levodopa (middle row), we found further enhancement of this BOLD pattern. Remarkably, the effect of reward magnitude on BOLD responses was attenuated and indeed eliminated after pretreatment with haloperidol (bottom row). There were no parametric effects of reward level for the earlier discrimination phase within a trial, for any pharmacological condition. Instead, the effect of reward level shown here under placebo, enhanced under levodopa, but eliminated under haloperidol, were specific to the time point for visual reward delivery.

Figure 5 plots the percent signal changes at reward delivery for reward minus nonreward trials, extracted from independently defined regions of interest (ROIs, see [14], Materials and Methods, and Discussion) contralateral to the rewarded index finger, for each drug group (separate rows in Figure 5). This reveals that our pharmacological manipulation of dopamine level influenced BOLD responses in PSC ROIs specifically as a function of the different financial levels achieved on reward trials (significant interaction between drug group and parametric reward-level; *F*_(2,27)_ = 10.27, *p*<0.001). Under placebo (top row in Figure 5), reactivation of contralateral PSC by visual reward feedback increased systematically with financial magnitude (*F*_(1,9)_ = 14.34, *p* = 0.004). Such an increase was also found under levodopa (*F*_(1,9)_ = 15.94, *p* = 0.003), with a trend towards a steeper slope than under placebo (*F*_(1,18)_ = 9.53, *p* = 0.07). Haloperidol, by contrast (see bottom row in Figure 5), completely eliminated the impact of a parametric reward level on PSC at reward delivery (*F*_(1,9)_ = 0.88, *p* = 0.37), with this flat function differing significantly from the linear increase under placebo in a direct comparison (*F*_(1,18)_ = 14.68, *p* = 0.001). All of these influences of reward level during visual reward delivery upon PSC were specific to the positive trials in which financial reward was delivered, with no effect of financial reward level on somatosensory cortex being found for nonrewarded trials instead, for all three groups here (all *p*>0.2). Thus, these effects are indeed due to the actual receipt of reward, rather than just general feedback on task performance.

### How Reward Enhances Behavioural Performance and Somatosensory Brain Activity

**Figure 6:**
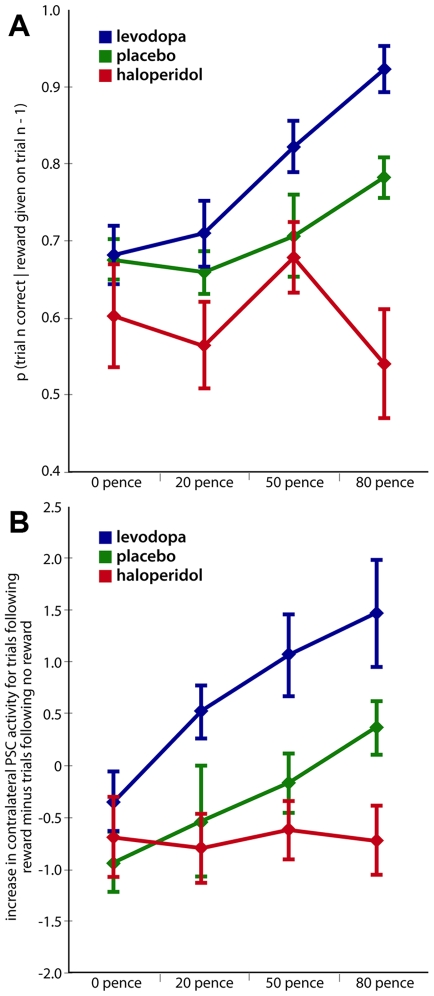
Trial-to-trial effects of receiving a higher reward. (A) shows for each drug group, the conditional probability of being correct in the somatosensory discrimination on a trial, given that the previous trial was rewarded. Group means are shown, with whiskers indicating s.e.m. Note the significantly higher conditional probability of being correct on the current trial following receipt of a higher reward on the previous trial for placebo (green line). This effect was even more pronounced for levodopa (blue line), but attenuated and indeed eliminated under haloperidol (red line). (B) ROI analysis of PSC (same ROIs as for Figure 5) reveals increased BOLD signal (group mean±s.e.m. shown), now during the somatosensory discrimination phase of trials, when those trials were preceded by actually receiving a higher reward at end of the prior trial, as compared with being preceded by a nonreward trial under that monetary level. Comparing placebo (green line) to levodopa (blue line), we found this trial-to-trial effect on the response of PSC was even more pronounced under the dopamine agonist, whereas it was attenuated and indeed eliminated by haloperidol (red line).

Our somatosensory decision task allows assessment of whether (higher) reward delivery on a given trial can enhance behavioural decisions and related PSC activity on the next trial [6]. Such trial-to-trial effects of reward delivery could explain why receiving higher rewards leads to better performance overall. Accordingly, we examined how dopamine may affect such trial-to-trial reward effects on somatosensory discrimination. We recently reported that the conditional probability of the next trial being correct after receiving a reward on the preceding trial is enhanced for higher rewards [6]. We now show that this behavioural effect is strongly modulated by dopamine, as manipulated here pharmacologically (interaction of reward level and the three drug groups, *F*_(2,27)_ = 7.6, *p* = 0.002). Under placebo (see green line in Figure 6A), the findings confirm our recent nonpharmacological study [6]. The beneficial impact of receiving reward on a given trial (*n*−1) for accurate performance on the subsequent trial (*n*) is stronger for higher reward levels (*F*_(1,9)_ = 14.62, *p* = 0.004). This reward level–dependent trial-to-trial effect was even more pronounced (*F*_(1,9)_ = 35.02, *p*<0.001) under levodopa (see blue line in Figure 6A), with a significantly steeper slope against the reward-level factor than for placebo (*F*_(1,18)_ = 7.49, *p* = 0.014). Levodopa also enhanced the overall trial-to-trial effect (pooled over reward level) relative to placebo (*F*_(1,18)_ = 4.68, *p* = 0.044). For haloperidol, by contrast (see red line in Figure 6A), the parametric increase in trial-to-trial performance (as a function of reward level obtained on the preceding trial) was completely eliminated (*F*_(1,9)_ = 0.102, *p* = 0.757) and hence reduced relative to the placebo group (*F*_(1,18)_ = 3.16, *p* = 0.09). Haloperidol likewise reduced the trial-to-trial effects relative to placebo when pooling across reward levels (*F*_(1,18)_ = 4.109, *p* = 0.05).

This aspect of our behavioural findings thus establishes dopamine-dependence for the enhancing effect of receiving a (higher) reward on the previous trial upon sensory decisions for the next trial, with this enhancement being even more pronounced under levodopa, but eliminated by haloperidol. Our final results confirm that such a dopamine-related trial-to-trial effect of reward level was not only present for sensory performance, but also impacted on the BOLD response of PSC during somatosensory discrimination for the next trial (interaction between drug group and parametric reward level, *F*_(2,27)_ = 4.48, *p* = 0.021, see Figure 6B). Using the same independently defined ROIs for PSC as previously (see Figure 4, and Materials and Methods for discussion on ROI selection), we found BOLD signal increases in PSC contralateral to the judged hand, now during the somatosensory stimulation/discrimination phase of a given trial, if a higher reward had actually been received on the previous trial. In line with our predictions [6], these trial-to-trial enhancements of PSC BOLD response by the level of reward actually received on the previous trial were present under placebo (*F*_(1,9)_ = 11.79, *p* = 0.007). Our new pharmacological manipulation revealed that these reward-dependent trial-to-trial enhancements of PSC were even more pronounced under levodopa (*F*_(1,9)_ = 23.58, *p* = 0.001; *F*_(1,18)_ = 3.16, *p* = 0.09 in direct comparison with placebo), but were completely abolished under haloperidol (*F*_(1,9)_ = 0.003, n.s.; *F*_(1,18)_ = 5.53, *p* = 0.03 in comparison with placebo). This pattern of results shows that pharmacologically manipulated dopamine level modulates the impact of reward for a given trial upon sensory performance (Figure 6A) and the response of PSC (Figure 6B) for the subsequent trial.

### How Reward Level and Dopaminergic Manipulations Influence Ventral Striatum and OFC

**Figure 7:**
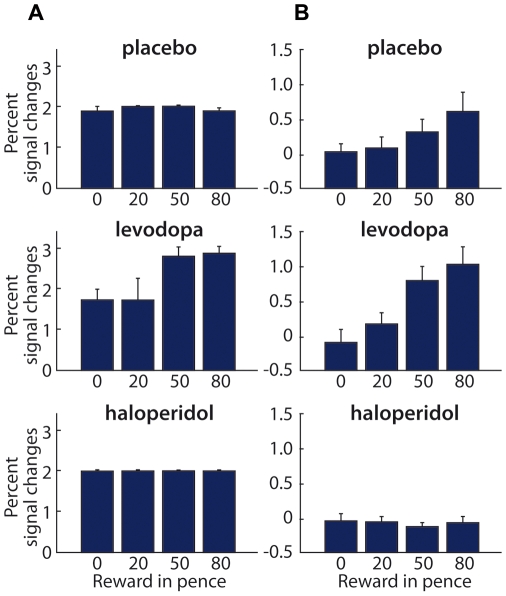
Percent signal changes in the ventral striatum corresponding to the time point of visual reward delivery (A) and the stimulation/discrimination point (B). (A) Mean BOLD signal changes (±s.e.m.) for the four financial reward-levels from the ventral striatum, at the later phase of reward delivery, with the three drug groups shown separately by row. These data were extracted from 5-mm spherical ROIs centred at *xyz* = −8, 12, −6, and 10, 18, −4, i.e., at the peaks for the fully orthogonal effect of reward minus nonreward feedback in our balanced factorial design. The plot shows that there is no strictly linear effect of reward level, but under levodopa, enhanced BOLD signal for the higher two reward levels is apparent. The placebo and haloperidol groups show flat functions, with this outcome under placebo replicating our recent nonpharmacological study [6]. (B) Mean signal changes (±s.e.m.) for the four financial reward levels from the same ventral striatum ROI, but now for the earlier stimulation/discrimination point, shown separately for the three drug groups. Under placebo (top row), there is a monotonic effect of increased potential reward level, replicating the same anticipatory effect found in the nonpharmacological study [6]. The middle row shows that this pattern is also present, and indeed enhanced, under levodopa. By contrast, haloperidol attenuates and indeed eliminates this effect.

Figure 7 plots the BOLD responses in ventral striatum for each financial reward level (OFC showed comparable signal changes for these comparisons). At the point of reward delivery (see Figure 7A), the ventral striatum showed a significant reward-by-drug interaction (*F*_(2,27)_ = 5.59, *p* = 0.009, for ventral striatum, see Figure 7A; and *F*_(2,27)_ = 5.26, *p* = 0.012, for OFC). At this time point, BOLD responses under levodopa showed an impact of reward versus nonreward for ventral striatum (*F*_(1,9)_ = 5.99, *p* = 0.03) that was enhanced relative to placebo (*F*_(1,18)_ = 5.57, *p* = 0.03) and likewise for OFC (*F*_(1,9)_ = 7.49, *p* = 0.02; levodopa vs. placebo: *F*_(1,18)_ = 3.51, *p* = 0.07).

However, the effects of reward delivery on ventral striatum and OFC differed from those observed in PSC (cf. Figure 5). In the placebo group, neither striatum (*F*_(1,9)_ = 0.01, *p* = 0.91) nor OFC (*F*_(1,9)_ = 3.6, *p* = 0.09) showed a significant increase in BOLD response with rising reward level at reward delivery point (consistent with [6]). Instead, the impact of reward level on striatum and OFC at reward delivery was only significant under levodopa (striatum: *F*_(1,9)_ = 5.99, *p* = 0.03; OFC: *F*_(1,9)_ = 7.49, *p* = 0.02). We found no parametric reward-level effect under haloperidol, neither in the striatum (*F*_(1,9)_ = 0.59, *p* = 0.46; haloperidol vs. placebo: *F*_(1,18)_ = 0.002, *p* = 0.96) nor in OFC (*F*_(1,9)_ = 0.01, *p* = 0.9; haloperidol vs. placebo: *F*_(1,18)_ = 3.6, *p* = 0.07) at reward delivery.

During the earlier stimulation/discrimination phase, both these reward-related regions showed an interaction of drug and reward level (striatum: *F*_(2,27)_ = 3.98, *p* = 0.03; OFC: *F*_(2,27)_ = 3.42, *p* = 0.04, for correct minus incorrect trials, see Figure 7B). For this relatively early point in the trial, BOLD responses under placebo replicated our recent nonpharmacological study [6] in showing a monotonic effect of increased anticipated reward level for ventral striatum (*F*_(1,9)_ = 5.12, *p* = 0.05) and OFC (*F*_(1,9)_ = 7.38, *p* = 0.02), in advance of actual reward delivery. This pattern was also found under levodopa (striatum: *F*_(1,9)_ = 7.03, *p* = 0.02; OFC: *F*_(1,9)_ = 6.03, *p* = 0.03), but it was attenuated, and indeed eliminated, under haloperidol (striatum: *F*_(1,9)_ = 0.1, *p* = 0.75; placebo vs. haloperidol: *F*_(1,18)_ = 4.75, *p* = 0.04; OFC: *F*_(1,9)_ = 0.02, *p* = 0.87; placebo vs. haloperidol: *F*_(1,18)_ = 6.55, *p* = 0.02).

Our data indicate that ventral striatum and OFC, both classic reward-related regions, also show a pattern of dopaminergic reward-related modulation, but this pattern differed from that seen in PSC. During the reward delivery phase, an influence of reward level was observed in ventral striatum and OFC only under levodopa (not during placebo, as in PSC). However, reward level affected BOLD signal in ventral striatum and OFC during the earlier stimulation/discrimination phase (where no effects was seen in PSC). Thus, the classic reward-related areas in ventral striatum and OFC mostly showed anticipatory effects of reward level at the earlier stimulation/discrimination point, whereas PSC was only influenced significantly by reward level at the later reward delivery phase (see above and Figures 4 and 5). Nonetheless, all of these effects were typically enhanced by levodopa but eliminated by haloperidol.

## Discussion

How the brain harnesses reward-related information to control a wide range of overt behaviours [15],[16] is a central topic in decision neuroscience. Much recent discussion concerns the likely dopaminergic mediation of such effects [1]. An emerging new question is whether reward may influence early sensory processing [6],[17],[18], and if so, whether these influences are dopaminergically mediated.

Here, we establish a dopamine dependence for reward-based influences on human PSC in a sensory decision-making task. We found that participants pretreated with levodopa showed increased reward-related effects for both tactile decisions and for hemodynamic responses (or “reactivation”) in PSC at the point of reward delivery (see Figures 4 and 5). Haloperidol, in contrast, eliminated these influences of reward on somatosensory performance and on processing in PSC. This demonstrates that dopaminergic neural processes, enhanced by levodopa but attenuated by haloperidol, are involved in modulating the impact of reward upon activity and function for primary somatosensation. There was no parametric effect of reward level on PSC at the time point corresponding to the earlier discrimination phase for correct minus incorrect trials (see Figure S1, and Text S1). Instead, the effect of financial reward level was expressed only at the end of the trial, timelocked to positive reward delivery via visual feedback (see Materials and Methods for how these different phases of the trial were separated). This confirms that the effects on PSC (as seen in Figures 4 and 5) must reflect feedback due to reward receipt, rather than modulation of sensory processing during the stimulation, as observed during attention [19].

Dopamine is a key neurotransmitter implicated in incentive motivation [20], memory formation [21],[22], and reinforcement learning [23],[24]. Furthermore, dopamine release optimises response selection in skilled nonautomatic tasks [25] and improves cognitive function by enhancing information processing [26],[27] and attentional accuracy [28]–[30], possibly via suppression of background noise and enhancement of task-related signals [31]. However, there seems to be a trade-off between dopamine levels and performance since individuals with pathologically increased dopamine activity (i.e., schizophrenia) have reduced function of attentional and sensorimotor systems, and administration of strong dopamine antagonists in these patients ameliorates their deficits [32].

In humans, dopamine-mediated reward effects are well established in midbrain, ventral striatum, and OFC; these structures represent key components of the human reward system [10],[11] and were activated here. However, reward-sensitive areas are tightly interconnected with other cortical regions, including via thalamocortical loops [18], suggesting that a complex network of dopaminergic projections [33] can also affect processing in other brain areas, such as PSC [34],[35]. It is tempting to speculate that this interconnected architecture provides the basis for a pervasive influence of reward on a wide range of cognitive processes, and our present results appear consistent with this perspective, extending the range of influences to include PSC and sensory decisions.

When inspecting our data for effects of reward level on ventral striatum and OFC, we found dopamine-related anticipatory effects of monetary incentive arising early in the trial, during sensory processing and prior to reward delivery (Figure 7B). Note that these effects arose much earlier than a later effect on PSC, expressed solely when a reward was actually received (see Figure 5). The finding that ventral striatum and OFC were affected by reward level more in an anticipatory than outcome-related fashion may appear at odds with a number of studies showing reward outcome effects in these structures (e.g., [36],[37]). On the other hand, our findings are compatible with several other studies showing anticipatory reward effects in ventral striatum and OFC [38]–[41]. One possible explanation for our specific results may be that we used relatively low levels of reward on each trial (i.e., 0, 20, 50, or 80 pennies per correct trial), since other studies showing outcome-related effects in OFC and ventral striatum often used much larger amounts of monetary reward (e.g., 0 to $10 as in [42]). Furthermore, in our design, reward level was always explicitly signalled by a visual cue indicating the potential reward for the next blocked series of trials. The ventral striatum is known to encode not only reward prediction, like in our study during stimulation/discrimination period, see also [43], but also reward prediction errors, which reflect a difference between predicted and received reward level during feedback/outcome (see also [1],[2],[44],[45]). Thus, the explicit predictability of reward value in our task (signalled blockwise via a visual cue) may explain an absent reward effect at the outcome/feedback point in ventral striatum.

Taken together, our findings suggest that when reward outcome depends on a veridical sensory decision, reward signals that arise in putative reward regions (such as ventral striatum during stimulation/discrimination period here) can be propagated to early sensory systems that are critical for sensory judgements (in this case, the PSC). These reward-related modulations reflect the magnitude of reward actually received, and may thus provide a possible dopaminergic “teaching signal” based upon reward delivery. This suggests that dopamine-related interplay between striatum, OFC, and sensory cortex may allow incentive motivation and feedback to shape cortical responses [23], in line with the recent finding [46] that corticostriatal interactions during processing of incentive stimuli covary with the COMT val158met polymorphism, which is linked to higher synaptic dopamine levels.

Our present fMRI findings clearly establish that dopamine levels can affect reward-related influences on PSC. Future invasive neurophysiological studies in animals may shed further light on the fine-grained neural mechanisms and circuits involved in reward-related dopaminergic modulation of PSC function. Some aspects of our results already provide an initial step towards a mechanistic account for how reward can impact on somatosensory discrimination performance. Notably, we found that the “reactivation” in PSC by reward delivery at trial end influenced both performance and evoked somatosensory responses for the next trial (Figure 6; see also [6]). An important new finding here is that this trial-to-trial effect of reward outcome was also mediated by dopaminergic transmission, being enhanced by levodopa and abolished by haloperidol. These modulatory trial-to-trial effects of dopamine on somatosensory performance and cortical processing specifically depended on the financial level of reward received, and thus did not simply indicate some form of general “resetting” for the next trial [47]. Instead, our results suggest that these effects reflect a dopamine-mediated learning signal [48], fed back to task-specific primary sensory cortex [6],[17], that enhances the response of somatosensory cortex and somatosensory performance for the next trial, thereby leading to enhanced outcomes, and to the improvement in sensory decisions under higher rewards.

Our findings show that dopamine mediates a reward influence on early human sensory cortex in a sensory decision-making task. Recent invasive studies in rats [17],[18], and monkeys [4],[49],[50] had begun to incorporate reward considerations into mechanistic accounts for motor choice, and increasingly for perceptual decisions [5]. The present human study indicates that even basic sensory discriminations and the function of early sensory structures (here, PSC) are influenced by dopaminergic transmission [7]. Thus, dopamine-dependent reward signals arising in classic reward-related structures appear to be propagated back to early somatosensory cortex so as to shape basic sensory discrimination, leading to enhanced reward outcome. This raises the tantalising possibility that specific pharmacological manipulations (e.g., those affecting dopaminergic systems) might modulate reward-related brain processes for possible neuro-rehabilitation of sensory processing.

## Materials and Methods

### Experimental Schedule

Participants first practiced the somatosensory frequency-discrimination task in an initial session inside the scanner, but without functional images being collected. This practice session had the same length as the subsequent experiment, but we presented only 0-pence trials to avoid habituation to reward magnitudes. Participants were then removed from the scanner, and drugs were administrated in a placebo-controlled, double-blind, fully randomized design. Since levodopa reaches peak plasma concentration within 1 h after intake, whereas haloperidol peaks 3 h later, we followed a recently described method [16] to ensure that peak plasma concentration of both drugs coincided with fMRI (see Figure S2). Participant always received two pills; the first immediately after the practise session, and the second 3 h later. The main experiment involving scanning started 1 h after the participant received the second pill. If a participant was assigned to the placebo group, both pills contained placebo. In the levodopa group, the first pill contained placebo, the second 100 mg of levodopa. Accordingly, latency between levodopa administration and main experiment was 1 h, which is the time a single dose of 100 mg needs to reach peak plasma concentration [16]. In the haloperidol group, the first pill contained 2-mg haloperidol, the second pill placebo. Thus, latency between haloperidol intake and main experiment was 4 h, in which time haloperidol is known to reach peak plasma concentration [16]. This drug administration schedule thus ensured that the peak plasma concentration of both drugs was matched across participants, without the necessity for further pharmacokinetic characterisation.

### Event-Related Functional Magnetic Resonance Imaging

Thirty right-handed healthy participants gave written informed consent in accord with local ethics. Ten participants (seven male) were included in each group in a fully randomized, double-blind fashion (placebo: aged between 21 and 35 y, mean 27±5.3 y; dopamine: aged between 19 and 31 y, mean 26±3.5 y; haloperidol: aged between 20 and 33 y, mean 27±4.5 y). All participants were European students. All females took contraceptives and were not scanned during menses. All participants were first interviewed and examined by an experienced physician (B. P.) to exclude any psychiatric/neurological symptoms and history of significant drug use.

We used a 3T head-scanner (Magnetom Allegra; Siemens) to acquire functional and structural brain scans. For functional brain scans, we used a BOLD-sensitive gradient echo T2* weighted echo-planar imaging (EPI) sequence (TE = 30 ms, TR = 2.21 s, flip angle = 90°, in-plane resolutio*n* = 3×3 mm^2^, slice-thickness = 2 mm, interslice distance = 1 mm) optimized for fMRI studies of the orbitofrontal cortex (for further information, see [51]). One MRI scan (or volume) consisted of 34 oblique slices (transversal-coronal tilt: −10°) covering the whole cerebrum. During each fMRI session we acquired 875 volumes continuously.

After drug administration, volunteers underwent an fMRI experiment in which they repeatedly discriminated the frequency of two electrical stimuli, applied sequentially to the index finger (both index fingers were in fact stimulated twice in succession on each trial, but only one hand or the other was judged). Participants experienced the stimulation as a prickling and tingling sensation, and reported that their decision was based on comparing the speed or rhythm of the two stimuli. With each trial, participants first perceive a stimulus, hold it in working memory, and finally make a decision by comparing it with a second stimulus (see also [9],[52]). For a detailed description of the fMRI design and the stimuli used; see [6]. Participants signalled their judgment via a foot response, and received visual feedback indicating positive or negative reward outcome (for correct or incorrect trials, respectively) after a variable temporal delay (see Figure 1). This temporal separation, and other standard aspects of event-related fMRI (e.g., [53]), allowed separation of BOLD signals attributable to somatosensory encoding from those due to the subsequent visual reward outcome (see Materials and Methods, and also [6] for in-depth discussion). Thus, any somatosensory reactivations due to the visual reward delivery must reflect reward-related signals, not the initial processing or level of attention during somatosensory input. We examined the influence of dopaminergic manipulations on reward-related processes at four different monetary reward levels (0, 20, 50, or 80 pennies per correct trial). These reward levels were organised into miniblocks of four successive trials (see Figure 1). The onset of each miniblock was signalled by a distinct visual cue indicating the potential reward for each of the next four trials, and also whether the right or left index finger should be judged for all those trials. Thus, the participant knew both the financial stake and which hand to judge in advance of each miniblock. Apart from this miniblock structure, levels of rewards were randomly intermingled, as was judged side. Our design enabled us to examine dopaminergic dependence of reward-related influences on somatosensory judgments and on related brain activity, both for overall effects of reward (regardless of financial level), as well as (orthogonally) for the parametric impact of increased potential rewards (i.e., 0, 20, 50, or 80 pence per correct judgment).

For a high-resolution structural brain scan, which was acquired after the functional MRI session, we used an isotropic 3D spoiled gradient-recalled (SPGR) sequence with 107 sagittal-orientated slices covering the whole brain. The anatomical images across participants were used to calculate a mean group image. For initial spatial assignment of functional changes, parametric maps showing the group statistics were superimposed onto this mean structural image.

### Statistical Analyses

We used SPM5 software (http://www.fil.ion.ucl.ac.uk/spm/) to assess event-related BOLD responses [53]. During the first six volumes per session, BOLD signal reached steady state. These volumes were discarded from further analysis. The remaining 869 volumes entered realignment and unwarping to remove movement artefacts [54]. Volumes were then spatially normalized to the standard template of the Montreal Neurological Institute [55]. As for our recent nonpharmacological study [6], we smoothed volumes using a 10-mm (full-width half-maximum) isotropic, three-dimensional Gaussian filter, in accord with the standard SPM approach.

To assess reliability of effects across participants, we used random-effects SPM analysis. We report all brain regions that survived family-wise error (FWE)-corrected thresholds. We further assessed whether particular hemodynamic changes could specifically be attributed to PSC, by restricting the analysis to PSC in both brain hemispheres. For this, we used a cytoarchitechtonic computerized anatomical atlas [13] (see http://www.fz-juelich.de/inb/inb-3//spm_anatomy_toolbox) to create masks according to the broad definition of PSC as encompassing BA1, BA2, and BA3b, based on separate postmortem data [12],[13],[56]. This ROI definition by means of anatomy prevented any selection bias and hence potential artificial inflation of our ROI statistics [14].

We identified effects attributable to distinct events using distinct stick functions (convolved with the default HRF in SPM). These stick functions encoded the timing of tactile stimulation, or of later reward feedback for each trial. We also used a stick function timelocked to the actual pedal response on each trial, and a further stick function timelocked to the visual cue at the start of each miniblock. This meant that all event types were coded as distinct events, except that the successive pair of somatosensory stimuli on each trial was coded as a single composite event since they were not jittered relative to each other (see Figure 1). We further distinguished between event types depending on the rewarded side (right or left), reward magnitude, and whether the judgment was correct or not (rewarded vs. nonreward trials). Null trials provided an implicit baseline. To consider general half-life issues of the drugs (i.e., haloperidol and levodopa), we followed a recently described procedure (see above, and [16]) that allowed us to use a statistical model for our fMRI data without taking pharmacokinetic characterization of the different drugs into account.

It was important for our experimental design to distinguish brain activity attributable to somatosensory stimulation/discrimination, from that due to later visual feedback signalling reward presentation. Unlike previous event-related fMRI studies on reward effects, which were not tailored to distinguish reward anticipation from sensation effects (see, e.g., [41],[57]), trial phases in our experiment could be separated due to a combination of a jittered timing (3–5 s intervening; 6–8 s from first somatosensory input) plus the fact that not all trials were rewarded (only correct) and reward could reflect four different monetary levels. The critical regressors for our analysis were demonstrably uncorrelated and therefore independent/orthogonal (see also [6]). The actual correlations between critical regressors across all three groups (levodopa, haloperidol, and placebo) were as follows: for correct discrimination versus reward feedback: *r* = −0.02; and for incorrect discriminations versus no-reward feedback: *r* = −0.08 (see also [6]). These vanishingly small (insignificant, null) correlations allowed us to separate discrimination-related versus feedback-related activity changes with a standard event-related SPM analysis (see also [58]–[60] for similar use of standard methods for decorrelating regressors in fMRI analyses).

Given other recent results from pharmacological fMRI involving levodopa and haloperidol [16], and in accord with their established impact on central dopamine action, we expected *reduced* reward-related effects (if dopaminergic) in the haloperidol group, and *enhanced* reward effects in the levodopa group, relative to placebo. Accordingly, on the between-group level, we coded the groups as three successive steps (haloperidol, then placebo, then levodopa) for parametric contrasts in the general linear model (i.e., weighting them as “1,” “2,” “3”). Nonreward trials were equally coded for all three groups (weighted as “−2”).

For further analysis of trial-to-trial effects, in terms of whether performance was rewarded or not on the preceding trial at a particular monetary level, we had to eliminate the last trial from each miniblock from consideration of possible effects on the next trial, as a different miniblock instruction intervened.

Hypothesis-driven ROI analyses [6],[61] were implemented using 5-mm spheres centred the peak coordinates for the categorical reward versus nonreward feedback effect in PSC, contralateral to the judged side (i.e., at −36, −36, 60 for the right and at 36, −30, 48 for the left index finger, as shown in Figure 4 and Table S2b). Note that these ROIs were fully unbiased, being derived from a categorical contrast orthogonal to (and hence independent of) any parametric effect related to monetary level of reward. In the context of our fully balanced factorial design, this regressor orthogonality ensures unbiased ROI statistics [14].

We averaged the signal within these spheres and submitted the values to conventional tests for significance across participants. Note that tests for any one particular factor were applied to ROIs whose location had been defined orthogonally by an independent contrast, to avoid circularity or bias in ROI selection.

## Supporting Information

Figure S1 Percent signal changes in PSC during the early stimulation/discrimination phase. Data are from ROIs contralateral to the stimulated right (red) or left (green) index finger, shown separately in different rows for the three drugs groups (group mean±standard error of the mean [s.e.m.]). Unlike activation in PSC for the same ROIs at the later phase of visual reward delivery (cf. Figure 5), we found no significant parametric influence of reward level on BOLD responses in PSC during the earlier stimulation/discrimination phase.
(0.80 MB TIF)

Figure S2 Randomized double-blinded drug schedule. After a practise session (which had the same duration as the fMRI experiment, but did not vary financial reward level), the participant always received two pills; the first pill immediately after the practise session, the second pill 3 h later. The fMRI experiment started 1 h after the participant received the second pill. If a participant was assigned to the placebo group, both pills contained placebo. In the levodopa group, the first pill contained placebo, the second 100 mg of levodopa. In the haloperidol group instead, the first pill contained 2-mg haloperidol, the second pill placebo. We used this procedure to ensure peak plasma concentration of the drugs during the fMRI experiment; see Materials and Methods.
(0.94 MB TIF)

Table S1 Brain regions activated by tactile discrimination task (versus baseline). Cortical regions of both hemispheres involved in the somatosensory frequency-discrimination task (versus the implicit null-event baseline). Shown are Montreal Neurological Institute (MNI) coordinates and T-scores of peak voxels contra- or ipsilateral to the judged index finger, surviving *p*<0.05 family-wise error-corrected threshold. PFC, prefrontal cortex; PMC, premotor cortex; PPC, posterior parietal cortex; SMA, supplementary motor area; SSC/PV, secondary somatosensory cortex/parietal ventral cortex.
(0.05 MB DOC)

Table S2 Brain regions activated for reward relative to nonreward visual feedback. Cortical regions of either hemisphere activated during reward feedback relative to nonreward feedback, with higher activity for levodopa and lower activity for haloperidol are compared to placebo: (A) across both index fingers; or (B) for each index finger separately. Shown are the MNI coordinates, the T-scores, and the associated p-values (family-wise error corrected and uncorrected).
(0.04 MB DOC)

Text S1 The somatosensory task and how reward level and drugs influence PSC in the stimulation/discrimination phase.
(0.03 MB DOC)

## Abbreviations

BOLD: blood oxygen level–dependent
fMRI: functional magnetic resonance imaging
OFC: orbitofrontal cortex
PSC: primary somatosensory cortex
ROI: region of interest
