# Supplementary figures and images for: Influence of Dopaminergically Mediated Reward on Somatosensory Decision-Making
Source: PLoS Biol. 2009 Jul 28;7(7):e1000164. doi: 10.1371/journal.pbio.1000164 (PMC2709435; doi:10.1371/journal.pbio.1000164)

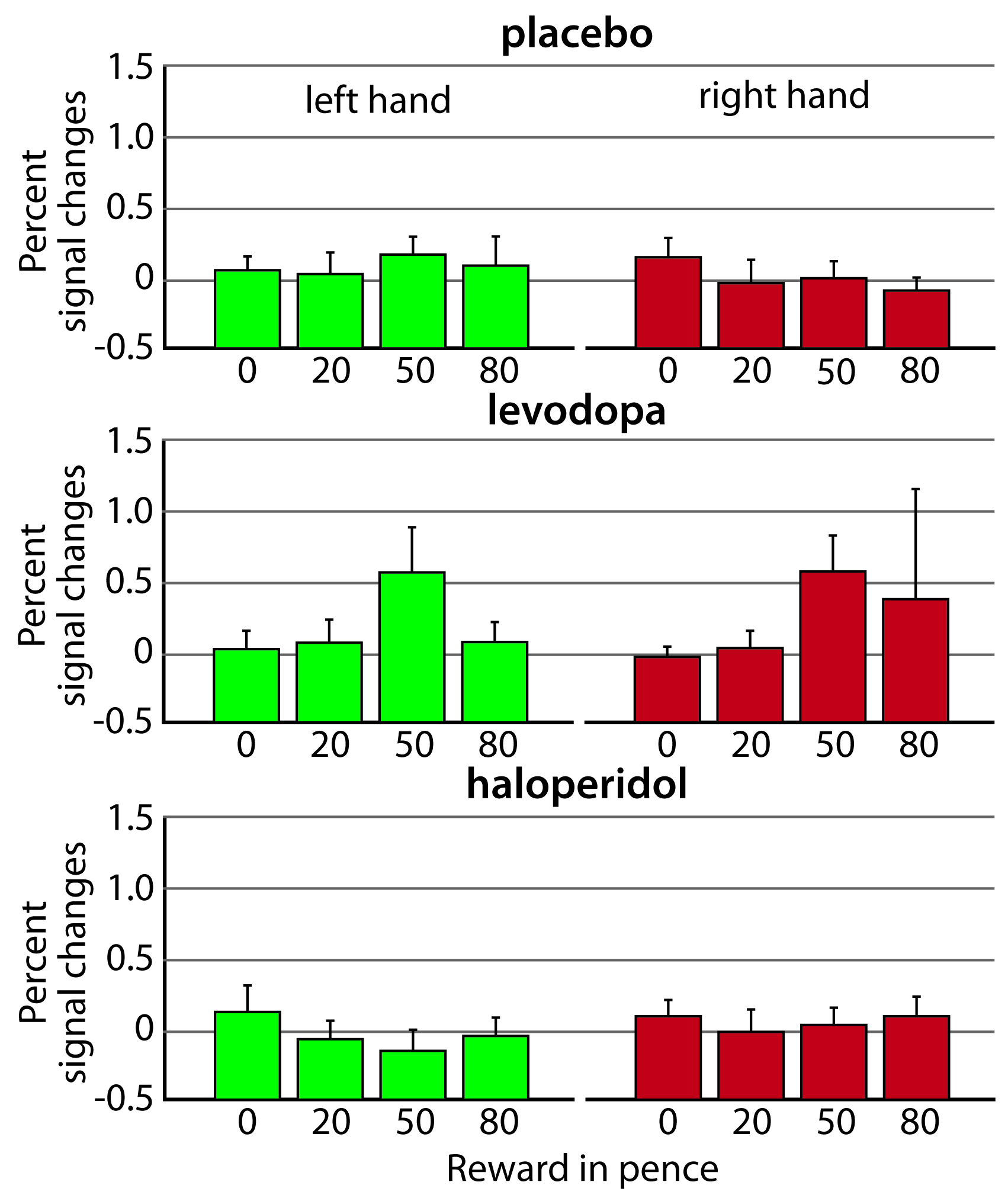

Supplement: Figure S1 — Percent signal changes in PSC during the early stimulation/discrimination phase. Data are from ROIs contralateral to the stimulated right (red) or left (green) index finger, shown separately in different rows for the three drugs groups (group mean±standard error of the mean [s.e.m.]). Unlike activation in PSC for the same ROIs at the later phase of visual reward delivery (cf. Figure 5), we found no significant parametric influence of reward level on BOLD responses in PSC during the earlier stimulation/discrimination phase. (0.80 MB TIF) [file pbio.1000164.s001.tif]

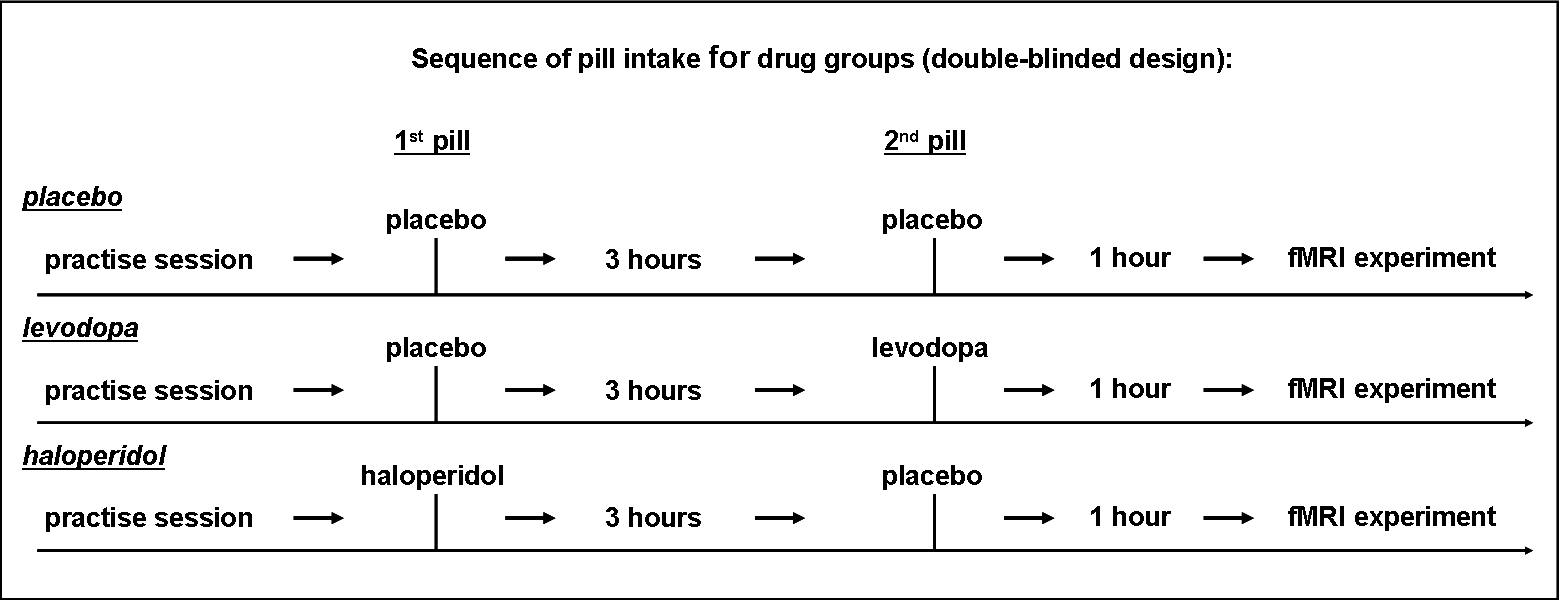

Supplement: Figure S2 — Randomized double-blinded drug schedule. After a practise session (which had the same duration as the fMRI experiment, but did not vary financial reward level), the participant always received two pills; the first pill immediately after the practise session, the second pill 3 h later. The fMRI experiment started 1 h after the participant received the second pill. If a participant was assigned to the placebo group, both pills contained placebo. In the levodopa group, the first pill contained placebo, the second 100 mg of levodopa. In the haloperidol group instead, the first pill contained 2-mg haloperidol, the second pill placebo. We used this procedure to ensure peak plasma concentration of the drugs during the fMRI experiment; see Materials and Methods. (0.94 MB TIF) [file pbio.1000164.s002.tif]
