# Supplementary material for: Influence of Dopaminergically Mediated Reward on Somatosensory Decision-Making
Source: PLoS Biol. 2009 Jul 28;7(7):e1000164. doi: 10.1371/journal.pbio.1000164 (PMC2709435; doi:10.1371/journal.pbio.1000164)
Supplement: Table S1 — Brain regions activated by tactile discrimination task (versus baseline). Cortical regions of both hemispheres involved in the somatosensory frequency-discrimination task (versus the implicit null-event baseline). Shown are Montreal Neurological Institute (MNI) coordinates and T-scores of peak voxels contra- or ipsilateral to the judged index finger, surviving p<0.05 family-wise error-corrected threshold. PFC, prefrontal cortex; PMC, premotor cortex; PPC, posterior parietal cortex; SMA, supplementary motor area; SSC/PV, secondary somatosensory cortex/parietal ventral cortex. (0.05 MB DOC) [file pbio.1000164.s003.doc]

**right index finger left index finger**

| **brain region** | **left hemisphere**  (contralateral to judged  right finger) | | | | **right hemisphere**  (ipsilateral to judged  right finger) | | | | **right hemisphere**  (contralateral to judged  left finger) | | | | **left hemisphere**  (ipsilateral to judged  left finger) | | | |
| --- | --- | --- | --- | --- | --- | --- | --- | --- | --- | --- | --- | --- | --- | --- | --- | --- |
|  | **MNI**  **coordinates** | | | **T**  **score** | **MNI**  **coordinates** | | | **T**  **score** | **MNI**  **coordinates** | | | **T**  **score** | **MNI**  **coordinates** | | | **T**  **score** |
|  | *x* | *y* | *z* |  | *x* | *y* | *z* |  | *x* | *y* | *z* |  | *x* | *y* | *z* |  |
| **PSC** | -40 | -42 | 60 | 9.2 | 54 | -26 | 50 | 10.54 | -50 | -34 | 54 | 9.08 | 54 | -26 | 50 | 11.92 |
| **SSC/PV** | -58 | -22 | 18 | 16.11 | 64 | -24 | 22 | 13.25 | -58 | -22 | 18 | 16.89 | 58 | -16 | 18 | 16.71 |
| **PPC** | -30 | -54 | 46 | 9.99 | 42 | -44 | 48 | 11.4 | -38 | -40 | 40 | 11.45 | 40 | -44 | 48 | 11.25 |
| **PMC** | -56 | 4 | 34 | 14.62 | 58 | 12 | 30 | 11.53 | -56 | 4 | 34 | 13.77 | 58 | 10 | 32 | 11.6 |
| **PFC** | -42 | 34 | 26 | 11 | 36 | 50 | 22 | 10.38 | -44 | 34 | 28 | 10.55 | 46 | 36 | 20 | 9.83 |
| **SMA** | -4 | 8 | 48 | 17.4 | 8 | 6 | 50 | 15.7 | -4 | 8 | 48 | 17 | 8 | 6 | 50 | 15.93 |
| **insula** | -44 | 2 | 6 | 15.76 | 56 | 12 | 2 | 13.91 | -44 | 2 | 6 | 16.26 | 54 | 12 | 4 | 14.88 |
| **caudate** | -12 | 10 | 6 | 10.29 | 10 | 10 | 6 | 10.13 | -14 | 12 | 0 | 10.91 | 10 | 12 | 8 | 9.56 |
| **thalamus** | -16 | -18 | 14 | 10.56 | 14 | -16 | 14 | 9.67 | -12 | -16 | 12 | 11.41 | 12 | -16 | 12 | 11.14 |
| **striatum** | -18 | 4 | -8 | 8.72 | 14 | 12 | -4 | 9.98 | -18 | 2 | -8 | 9.28 | 16 | 12 | -8 | 8.9 |
