# Supplementary material for: Influence of Dopaminergically Mediated Reward on Somatosensory Decision-Making
Source: PLoS Biol. 2009 Jul 28;7(7):e1000164. doi: 10.1371/journal.pbio.1000164 (PMC2709435; doi:10.1371/journal.pbio.1000164)
Supplement: Table S2 — Brain regions activated for reward relative to nonreward visual feedback. Cortical regions of either hemisphere activated during reward feedback relative to nonreward feedback, with higher activity for levodopa and lower activity for haloperidol are compared to placebo: (A) across both index fingers; or (B) for each index finger separately. Shown are the MNI coordinates, the T-scores, and the associated p-values (family-wise error corrected and uncorrected). (0.04 MB DOC) [file pbio.1000164.s004.doc]

**a)**

| **brain region** | **Left hemisphere** | | | | | **Right hemisphere** | | | | |
| --- | --- | --- | --- | --- | --- | --- | --- | --- | --- | --- |
|  | **MNI**  **coordinates** | | | **T** | **p-corrected/**  **p-uncorrected** | **MNI**  **coordinates** | | | **T** | **p-corrected/**  **p-uncorrected** |
|  | *x* | *Y* | *z* |  |  | *x* | *y* | *z* |  |  |
| **Ventral striatum** | -8 | 12 | -6 | 10.11 | <0.001/ 4.4*10^-16^ | 10 | 18 | -4 | 11.04 | <0.001/ 4.4*10^-16^ |
| **Orbitofrontal cortex** | -8 | 48 | -10 | 9.67 | 1.5*10^-11^ / 5.5*10^-16^ | - | - | - | - | - / - |
| **PSC** | -34 | -32 | 66 | 6.26 | 6.91*10^-6^ / 3.94*10^-9^ | 36 | -30 | 48 | 5.3 | 3.76*10^-4^ / 3.13*10^-7^ |

**b)**

| **brain region** | **reward versus no-reward feedback**  **for the right index finger** | | | | | **reward versus no-reward feedback**  **for the left index finger** | | | | |
| --- | --- | --- | --- | --- | --- | --- | --- | --- | --- | --- |
|  | **MNI**  **coordinates** | | | **T score** | **p-corrected/**  **p-uncorrected** | **MNI**  **coordinates** | | | **T score** | **p-corrected/**  **p-uncorrected** |
|  | *x* | *Y* | *z* |  |  | *x* | *y* | *z* |  |  |
| **PSC** | -36 | -36 | 60 | 3.99 | 0.018 / 6*10^-5^ | 36 | -30 | 48 | 4.58 | 0.003/ 6.36*10^-6^ |
