# Supplementary material for: Influence of Dopaminergically Mediated Reward on Somatosensory Decision-Making
Source: PLoS Biol. 2009 Jul 28;7(7):e1000164. doi: 10.1371/journal.pbio.1000164 (PMC2709435; doi:10.1371/journal.pbio.1000164)
Supplement: Text S1 — The somatosensory task and how reward level and drugs influence PSC in the stimulation/discrimination phase. (0.03 MB DOC) [file pbio.1000164.s005.doc]

**Supporting Information**

**The brain network involved in the somatosensory task**

To identify the overall brain network involved in the somatosensory task, we first compared tactile-task events in all subjects against baseline activity. This baseline activity was measured by ‘null trials’ [1], i.e., trials without tactile stimulation or feedback. In line with previous studies [2-5], this comparison revealed activation for the somatosensory task in primary somatosensory cortex (PSC), and secondary somatosensory cortices/parietal ventral cortex; as well as in prefrontal cortex (PFC), supplementary motor area (SMA), premotor cortex (PMC), posterior parietal cortex (PPC), insula, caudate nucleus, and striatum in both hemispheres (see Table S1). We found no differences in these BOLD responses due to the overall tactile-task between the drug-groups (i.e., not for placebo vs. levodopa, placebo vs. haloperidol, nor levodopa vs. haloperidol).

We also compared BOLD responses for correct trials with those for incorrect trials (for all subjects, within each group and between drug groups), during the earlier stimulation/discrimination phase of each trial (rather than the later visual reward feedback period). In agreement with previous findings [3], we observed no differential effect for this categorical comparison in primary somatosensory cortex, nor within other brain areas for the stimulation/discrimination phase. The main paper reports the significant results found for reward versus non-rewarded trials at the later visual feedback period instead (e.g. see Figures 3 and 4 in main paper).

**Reward-level and drugs do not influence primary somatosensory cortex in the stimulation/discrimination phase**

In the stimulation/discrimination phase, for, correct minus incorrect trials we found no interaction between drugs and reward-level (F_(2,27)_=1.52, p=.23; Fig. S1). The financial level did not significantly affect BOLD responses in primary somatosensory cortex for this phase within a trial in any group (all p>.2, see Table S1, and Fig. S1). This lack of reward-level effects in the stimulation/discrimination phase for placebo is in agreement with other recent findings by our group [3], and as in that study this shows that the reward related re-activation of somatosensory cortex cannot merely reflect sensory attention during the somatosensory stimuli (see also Materials and Methods in main paper). As in our recent non-pharmacological study [3], we again found that the significant effect of reward-level on primary somatosensory cortex (found under placebo and enhanced under levodopa) was specifically expressed at trial end, at the time-point of reward-delivery (see Fig. 5 in main paper), not during somatosensory stimulation (Fig. S1). This led to significantly greater reward-level effects at reward-delivery than in the stimulation/discrimination phase (under placebo: F_(1,18)_=14.59, p=.001, and under levodopa: F_(1,18)_=9.15, p=.007). This confirms that the effect on primary somatosensory cortex shown in main Figures 4 and 5 must reflect (visual) reward-delivery, rather than other factors such as sensory attention during the stimulation. Under haloperidol there was no effect of financial reward level at any phase (see bottom row of Fig. 5 in main paper and also of Fig. S1, F_(1,18)_=.087, p=.771, n.s).

**References**

1. Henson RNA (2003) Analysis of fMRI time series. In: Frackowiak RSJ, Friston KJ, Frith C, Dolan RJ, Price CJ, et al., editors. Human Brain Function. New York: Academic Press: pp. 793-822.

2. Romo R, Salinas E (2003) Flutter discrimination: neural codes, perception, memory and decision making. Nat Rev Neurosci 4: 203-218.

3. Pleger B, Blankenburg F, Ruff CC, Driver J, Dolan RJ (2008) Reward facilitates tactile judgments and modulates hemodynamic responses in human primary somatosensory cortex. J Neurosci 28: 8161-8168.

4. Wang X, Merzenich MM, Sameshima K, Jenkins WM (1995) Remodelling of hand representation in adult cortex determined by timing of tactile stimulation. Nature 378: 71-75.

5. Golaszewski SM, Siedentopf CM, Koppelstaetter F, Fend M, Ischebeck A, et al. (2006) Human brain structures related to plantar vibrotactile stimulation: a functional magnetic resonance imaging study. Neuroimage 29: 923-929.
